# MHC-identical and transgenic cynomolgus macaques for preclinical studies

**DOI:** 10.1186/s41232-018-0088-3

**Published:** 2018-11-22

**Authors:** Hirohito Ishigaki, Takashi Shiina, Kazumasa Ogasawara

**Affiliations:** 1 Division of Pathology and Disease Regulation, Department of Pathology, Shiga University of Medical Science, Setatsukinowa, Otsu, Shiga 520-2192 Japan; 2 Department of Molecular Life Science, Division of Basic Medical Science and Molecular Medicine, Tokai University School of Medicine, 143 Shiomokasuya, Isehara, Kanagawa 259-1193 Japan; 3 Research Center for Animal Life Science, Shiga University of Medical Science, Setatsukinowa, Otsu, Shiga 520-2192 Japan

**Keywords:** Cynomolgus macaque, Transplantation, MHC-homozygous, iPSC, Genetically modified macaque

## Abstract

Cynomolgus macaques are useful experimental animals that are physiologically and genetically close to humans. We have developed two kinds of experimental usage of cynomolgus macaque: transplantation and disease models. First, we identified certain major histocompatibility complex (MHC) haplotypes including homozygotes and heterozygotes in cynomolgus macaques native to the Philippines, because they have less polymorphism in the MHC than that in other origins such as Vietnam and Indonesia. As a preclinical model of the induced pluripotent stem cell (iPSC) stock project, we established iPSCs from various types of MHC homozygous macaques, which were transplanted into compatible MHC heterozygous macaques, the iPSC stock project was experimentally shown to be effective. Second, to obtain disease models of cynomolgus macaques for studies on regenerative medicine including cell therapies, we established two kinds of genetic technology to modify cynomolgus macaques: transgenic technology and gene editing technology using CRISPR-Cas9. We will establish disease models, such as Alzheimer’s disease and progeria (Werner syndrome). In future, we will distribute the MHC-identical cynomolgus monkeys and genetically modified macaques to researchers, especially those engaging in regenerative medicine.

## Background

**Fig. 1:**
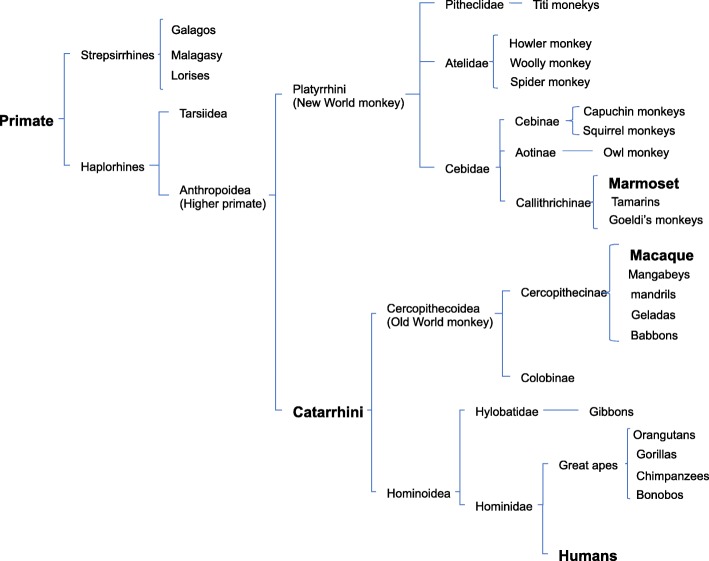
Taxonomic classification of extant primates. Macaques are closer to humans in the taxonomic classification. Hominoidea cannot be used for experiments for ethical reasons. Cercopithecoidea (Old World monkeys) is available as experimental animals that are closer to humans than other primates to humans. This figure combines Fig 4.23, Fig 5.28, Fig 6.22, and Fig 7.17 in reference 6

Non-human primates are useful experimental animals for preclinical experiments because they have almost the same genes and proteins as those in humans, resulting in almost the same immunity and metabolism [1–5]. Therefore, experimental results obtained by using non-human primates are more reliable than those obtained by using other mammalian species for extrapolation of the results to humans. As shown in Fig. 1, which is modified from reference 6, primate are classified into prosimians and anthropoids. Generally, prosimians live in trees and anthropoids live on the ground. Anthropoids consist of new world monkeys and old world monkeys, which include marmosets and macaques, respectively [6]. Macaques include Japanese snow monkeys, rhesus macaques, and cynomolgus macaques (Fig. 1). Of note, we are not able to use hominoids for biological experiments with an invasive procedure for ethical reasons. In our facility, we maintain breeding of about 700 cynomolgus macaques as experimental animals and we have performed infectious experiments of biosafety level 3 (BSL3) using several hundred cynomolgus macaques because the size of a cynomolgus macaque is about half of a Japanese monkey or rhesus macaque and thus feeding or drug administration for cynomolgus macaques is only about half of that required Japanese monkey and rhesus macaque.

Marmosets and cynomolgus macaques both have their own advantages. Marmosets mature in about one and a half years, whereas sexual maturation of cynomolgus macaques requires 4 years. Therefore, marmosets are easily established disease models due to their shorter life span. On the other hand, marmosets are too small (about 300 g) to use for organ transplantation, compared with cynomolgus macaques (about 5000 g). Furthermore, in experiments using cynomolgus macaques, we can use antibodies against human molecules since cynomolgus macaques are phylogenically closer than marmosets to humans (Fig. 1). We have therefore focused on the use of cynomolgus macaques as experimental animals.

### MHC and MHC haplotypes of cynomolgus macaque

Immune cells, especially T cells, recognize and attack cells and organs of non-selves carrying another major histocompatibility complex (MHC). Thus, in transplantation experiments, grafts expressing matched MHC molecules are required. Generally, grafts from MHC homozygous donors are immunologically acceptable to MHC-matching heterozygous recipients, while grafts containing mismatched-MHC are recognized and immunologically rejected by recipient immune cells.

Since MHC homozygous macaques are essential in experiments as donors for transplantation, we searched for such cynomolgus macaques in various polulations. Cynomolgus macaques are originally native to South-East Asia. In the age of discovery, Europeans brought macaques to Mauritius Island by ship as companion animals and then the macaques bred after escaping [7]. Therefore, Mauritian macaques typically contain only about 10 MHC haplotypes, which is lowest polymorphism in MHC of cynomolgus macaques [8–14]. Mauritian macaques are suitable for transplantation experiments, but unfortunately we are not able to use Mauritian macaques because of import restrictions by Japanese government due to the Ebola hemorrhage fever in Africa [15]. Accordingly, we use Philippine macaques in transplantation experiments because Philippine macaques have lower MHC polymorphism than those of Vietnam and Indonesia macaques, but their genetic diversity is maintained very well as same as the other populations [16]. After MHC typing of several thousands of macaques, we found some MHC haplotype homozygous individuals in the Philippine population.

**Fig. 2:**
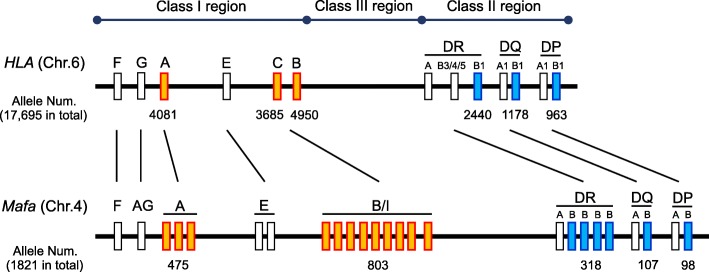
Comparative genome maps of representative MHC genes in humans and cynomolgus macaques. HLA and *Mafa* mean human and cynomolgus macaque MHCs, respectively. Orange boxes indicate classical class I genes, *HLA-A, HLA-B, HLA-C* and their *Mafa* orthologs (*Mafa-A, Mafa-B* and *Mafa-I*), in the class I sub-region, and blue boxes indicate classical class II genes, *HLA-DR, HLA-DQ, HLA-DP* and their *Mafa* orthologs (*Mafa-DR, Mafa-DQ* and *Mafa-DP*), in the class II sub-region. Numbers under boxes and in parentheses indicate allele numbers reported by the IPD-IMGT/HLA database release 3.31.0 in January 2018 in humans (Available from: https://www.ebi.ac.uk/ipd/imgt/hla/) and the IPD-MHC database release 3.0.0.1 in February 2018 in cynomolgus macaques (Available from: https://www.ebi.ac.uk/ipd/mhc/group/NHP/allele/Mafa)

**Fig. 3:**
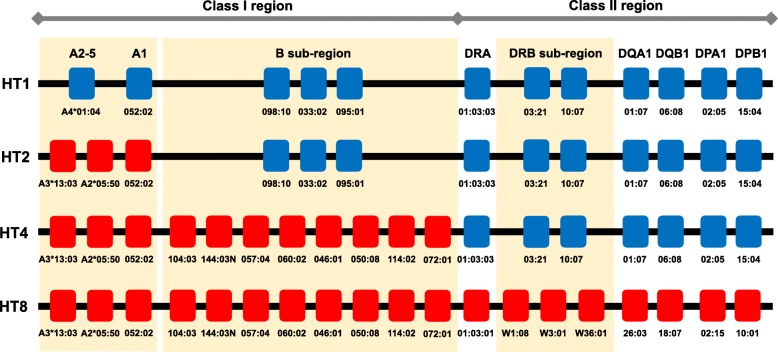
Representative *Mafa* haplotypes in Philippine population. Blue and red indicate *Mafa* alleles originating from the HT1 haplotype and HT8 haplotype, respectively. Yellow background indicates *Mafa* gene segments that are organized by CNV

Human and cynomolgus macaque MHCs are called as HLA and *Mafa*, respectively, and they contain a lot of genes related to immune response, which code molecules on the cell surface recognized by recipient immune cells. The HLA and *Mafa* regions are located on chromosome 6 in humans and chromosome 4 in cynomolgus macaques and they are divided into three sub-regions, class I, class II, and class III. Classical class I genes, *HLA-A*, *HLA-B*, *HLA-C*, and their *Mafa* orthologs (*Mafa-A*, *Mafa-B*, and *Mafa-I*), are included in the class I sub-region, and classical class II genes, *HLA-DR*, *HLA-DQ*, *HLA-DP*, and their *Mafa* orthologs (*Mafa-DR*, *Mafa-DQ*, and *Mafa-DP*), are included in the class II sub-region (Fig. 2). Although copy number variation (CNV) is usually observed in *Mafa-A*, *Mafa-B*, *Mafa-I*, and *Mafa-DR* genes in the *Mafa* region, the genomic structure of the *Mafa* region is similar to that of the HLA region [16, 17]. In macaques native to the Philippines, we have so far identified at least 20 *Mafa* haplotypes (HTs). Of them, HT1 and HT8 haplotypes have completely different *Mafa* alleles on all *Mafa* loci, and macaques that have these haplotypes are mutually used as *Mafa*-mismatched controls (Fig. 3). In contrast, HT2 and HT4 haplotypes are recombinants of HT1 and HT8 haplotypes (Fig. 3). Using macaques with these haplotypes might reveal which of the classes, class I and class II, works as the major factor in rejection.

### iPSCs of MHC identical cynomolgus macaques as the preclinical model of the iPS stock project in Japan

In the induced pluripotent stem cell (iPSC) stock project, HLA haplotype homozygous iPSCs are collected from healthy donors for treatment of HLA-matched patients. Transplantation of grafts or cells differentiated from self iPSC of patients has three major problems: high cost, time-consuming to process for preparation of differentiated cells, and preserving a genetic disorder if a patient has a genetic disorder. Pre-established (ready-made) HLA homozygous iPSCs are expected to resolve these problems. To investigate the efficacy of pre-established MHC homozygous iPSCs, we established a macaque transplantation model system in which differentiated cells from iPSCs with homozygous *Mafa* haplotypes prepared by Okita in Center for iPS Cell Research and Application (CiRA) were transplanted into *Mafa*-matched cynomolgus macaques. *Mafa*-matching means identical alleles in MHC class I genes (*Mafa-A, Mafa-B*) and MHC class II genes (*Mafa-DR*, *Mafa-DQ*, and *Mafa-DP*), and *Mafa*-mismatching involves different alleles in the MHC class I genes and the MHC class II genes. In these experiments, macaques that have the HT1 haplotype are mainly used because of their abundant population. Furthermore, to maintain a requisite number of *Mafa*-matched monkeys, we prepared *Mafa*-homozygous macaques using intracytoplasmic sperm injection (ICSI) [18]. Namely, *Mafa* homozygous spermatocytes were injected into *Mafa* heterozygous oocytes using a microinjector. We have so far produced four *Mafa* homozygous and more than 10 *Mafa* heterozygous macaques. Consequently, we have established a macaque transplantation system.

Our macaque transplantation system was used for transplantation of differentiated iPSCs, including retinal pigment epithelium [19], dopamine-producing cells [20], and sheets of cardiomyocytes [21] and cardiomyocytes [22]. The differentiated cells from *Mafa* homozygous iPSCs were functional in vivo and that minimal rejection was observed after transplantation. In addition, doses of immune suppressive drugs were reduced in *Mafa*-matched allogenic transplantation compared with that in *Mafa*-mismatched allogenic transplantation [19–22].

Recently, universal donor cells (UDCs), which are pluripotent stem cells without any MHC class I molecule expression except for MHC-E, were reported [23, 24]. The differentiated cells derived from UDCs are not recognized by host T cells, because they do not express any MHC molecules. Furthermore, they can escape from natural killer (NK) cell attack due to the expression of MHC-E, which is the ligand of the inhibitory receptor of NK cells, NKG2A/CD94 complex. Although transplantation of differentiated cells derived from UDCs seems to be free from rejection in allogenic transplantations, MHC class I expression is clinically important for T cell function such as graft versus leukimia (GVL) and reconstruction of immune systems after transplantation in the bone marrow transplantation of leukemia patients. In this regard, MHC homozygous iPSCs are also useful for regenerative medicine.

### Disease models of cynomolgus macaques: Transgenic cynomolgus macaques

Disease models of cynomolgus macaques are necessary for preclinical experiments in medical science including regenerative medicine. To establish disease models, we have established two kinds of genetically modified technology in cynomolgus macaques: transgenic technology and gene editing techonology. Genetically modified macaques are thought to be useful for preclinically testing new therapies against intractable diseases. In order to establish a method to produce transgenic cynomolgus macaques, we first prepared a green fluorescence protein (GFP) transgenic macaque by injecting a lentivirus encoding GFP into mature oocytes [25]. Using this method, we have produced macaques with Alzheimer’s disease highly expressing amyloid-β precursor protein (APP). The macaques have not yet expressed symptoms, because they are around one year old. We continuously investigate behavior of the macaques and are going to test an agent for early detection of Alzheimer’s disease [26].

By using genome editing with CRISPR-Cas9, we produced progeria (Werner syndrome). Mutation and inactivation of the *WRN* gene cause Werner syndrome, an autosomal recessive disease characterized by premature aging, elevated genomic instability, and increased cancer incidence [27, 28]. Knock-out of the *WRN* gene in mice did not fully reproduce the disease phenotype, because mice have long telomeres and a nucleolar localization signal of the WRN protein is absent in mice, unlike in humans and macaques. The *WRN* gene in cynomolgus macaques is similar to that in humans [29, 30]. A progeria model of cynomolgus macaques would be useful for research of atheroscrelosis, cancer, and diabetes mellitus.

The establishment of a monkey cancer model is necessary for preclinical experiments on cancer therapies. However, spontaneous neoplasms and malignant tumors in cynomolgus monkeys are uncommon [31]. To establish a monkey cancer model, we transplanted cancer cell lines of an MHC homozygous monkey established by transducing oncogenes into monkeys carrying the matched Mafa haplotype in one of the chromosomes. Therefore, MHC-matched cynomolgus macaques are urgently required. We established malignant (cancer) cells, such as embryonal carcinoma and glioblastoma, artificially induced from MHC homozygous iPSCs by injecting oncogenes. These malignant cells showed similar pathological features in NOG mice to those seen in humans. The embryonal carcinoma cells expressed AFP, OCT3/4, PLAP, and CD30, and glioblastoma cells did S100, GFAP, and Ki67. These cells were rejected by host immune cells even in MHC-matched heterozygous hosts because of the cancer antigen of glucose-regulated protein 94 (GRP94), which is one of chaperon proteins in ER and is expressed on the surface of cancer cells during cancerization [32]. In the process of making this cancer cell transplantation model, we recognized the importance of cancer immune surveillance and immune editing during cancer promotion. Thus, the host immune cells attack cancer cells before forming a tumor mass. Namely, cancer cells reduce their immunogenicity to escape the immune attacked during their development in vivo. Consequently, spontaneous cancer cells in patients might have been immune-edited to be less antigenic. Thus, we are trying to make a genetically modified cancer model in cynomolgus macaques in which tumors might have low antigenicity by immune editing after cancer immune surveillance.

## Conclusions

Cynomolgus macaques are useful experimental animals that are physiologically, biologically, and genetically closer to humans than are the other experimental animals. MHC-identified cynomolgus macaques native to the Philippines are necessary for a transplantation study in a preclinical setting. We identified four kinds of MHC homozygous cynomolgus macaques and established the iPSCs from them. We are also preparing a sufficient number of MHC-matched heterozygous macaques with ICSI. This MHC-matched macaque system has been used in transplantation studies and has been shown to be useful.

We have also established techniques of genetic modification in cynomolgus macaques with which disease models such as models of Werner syndrome and Alzheimer’s disease have been established. Since the establishment of a cynomolgus macaque cancer model is one of the important projects in the field of cancer science, we are going to develop a cancer model of MHC-identical macaques using techniques for genetic modification.

In the future, to easily use these monkeys for preclinical research, we will distribute the MHC-identical cynomolgus macaques and genetically modified macaques to researchers, especially those engaging in regenerative medicine.

## Abbreviations

CiRA: Center for iPS Cell Research and Application
GRP94: Glucose-regulated protein 94
GVL: Graft versus leukemia
HLA: Human leukocyte antigen
ICSI: Intra cytoplasmic sperm injection
iPSC: Induced pluripotent stem cell
MHC: Major histocompatibility complex
NK: Natural killer
UDC: Universal donor cell
